# Bowen’s Disease of the Labia Majora: A Rare Case

**DOI:** 10.7759/cureus.78542

**Published:** 2025-02-05

**Authors:** Manish Kumar, Zeenat S Imam, Shashi S Pawar, Sunny Singh, Prabhjot S Ahluwalia, Sudhir K Singh

**Affiliations:** 1 Surgical Oncology, Indira Gandhi Institute of Medical Sciences, Patna, IND; 2 Pathology, Indira Gandhi Institute of Medical Sciences, Patna, IND

**Keywords:** bowen’s disease, in situ, labia majora, rare site, squamous cell carcinoma

## Abstract

Bowen’s disease is a non-melanocytic in situ squamous cell carcinoma of the epidermis. Having a multifactorial etiology, its incidence has been found to be the highest in Caucasians. It commonly occurs as a solitary lesion in the elderly on the photoexposed areas of the skin, mainly in the head and neck region. We present a case of a single, pruritic, oozy ulcerated lesion in the left upper labia majora in a 32-year-old female for the last six months who had undergone various treatments before presenting to our institute. Excision followed by histopathological examination led to the diagnosis of Bowen’s disease. Our case focuses on the potential of Bowen’s disease to mimic common dermatological lesions, and therefore a high degree of suspicion assisted by histopathological examination is essential for early and rapid diagnosis, along with a multimodality approach to management.

## Introduction

Bowen’s disease is defined as a non-melanocytic, premalignant skin tumor. It is an intraepithelial carcinoma of the epidermis occurring in both the exposed and unexposed areas, with the head, neck, and lower limbs being the most common sites [1]. Sometimes, it can affect the groin area in males and the vulval area in females. It is most commonly seen in elderly light-skinned people without any sex predilection. It is extremely rare below the age of 30 years [2]. The etiology is multifactorial with major risk factors being fair skin, photosensitive individuals, exposure to ultraviolet (UV) light, decreased immune status, and human papillomavirus infections [3]. Histopathological examination is the gold standard modality for diagnosis.

Multiple treatment modalities are available; however, the choice of treatment is guided by the efficacy, location, size, focality, quantity of lesions, thickness, availability of treatment, patient’s age, immunity, comorbidities, medication history, clinician’s expertise, cosmetic outcome, and patient’s preference. In extragenital lesions, the risk of progression into an invasive carcinoma is 3%-5%, and about 10% is in genital lesions [4,5]. We report an unusual case of Bowen’s disease presenting as a chronic single ulcerative lesion of the left upper labia majora.

## Case presentation

**Figure 1:**
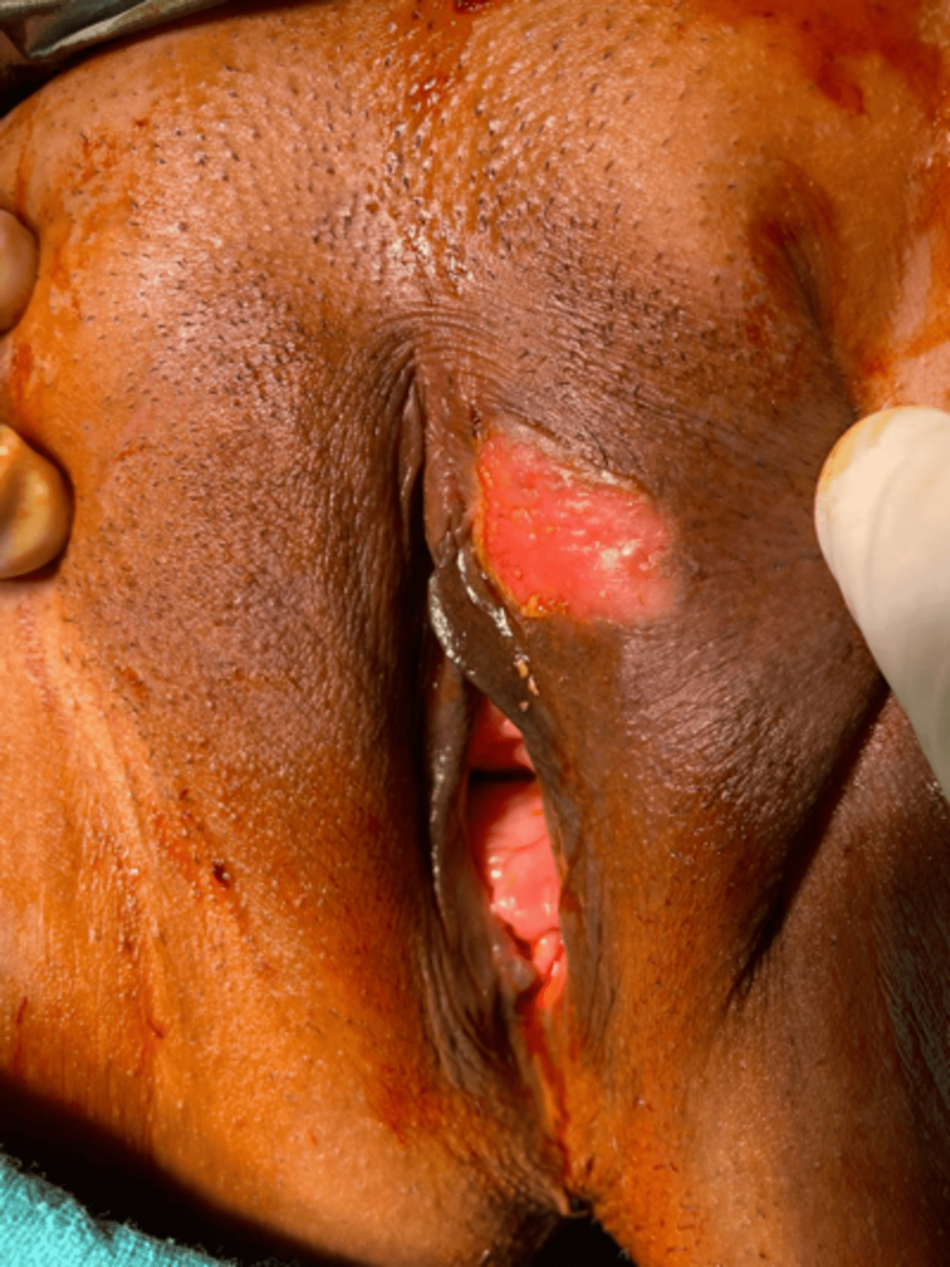
Preoperative status A solitary ulcerative lesion was seen at the left labia majora upper part.

**Figure 2:**
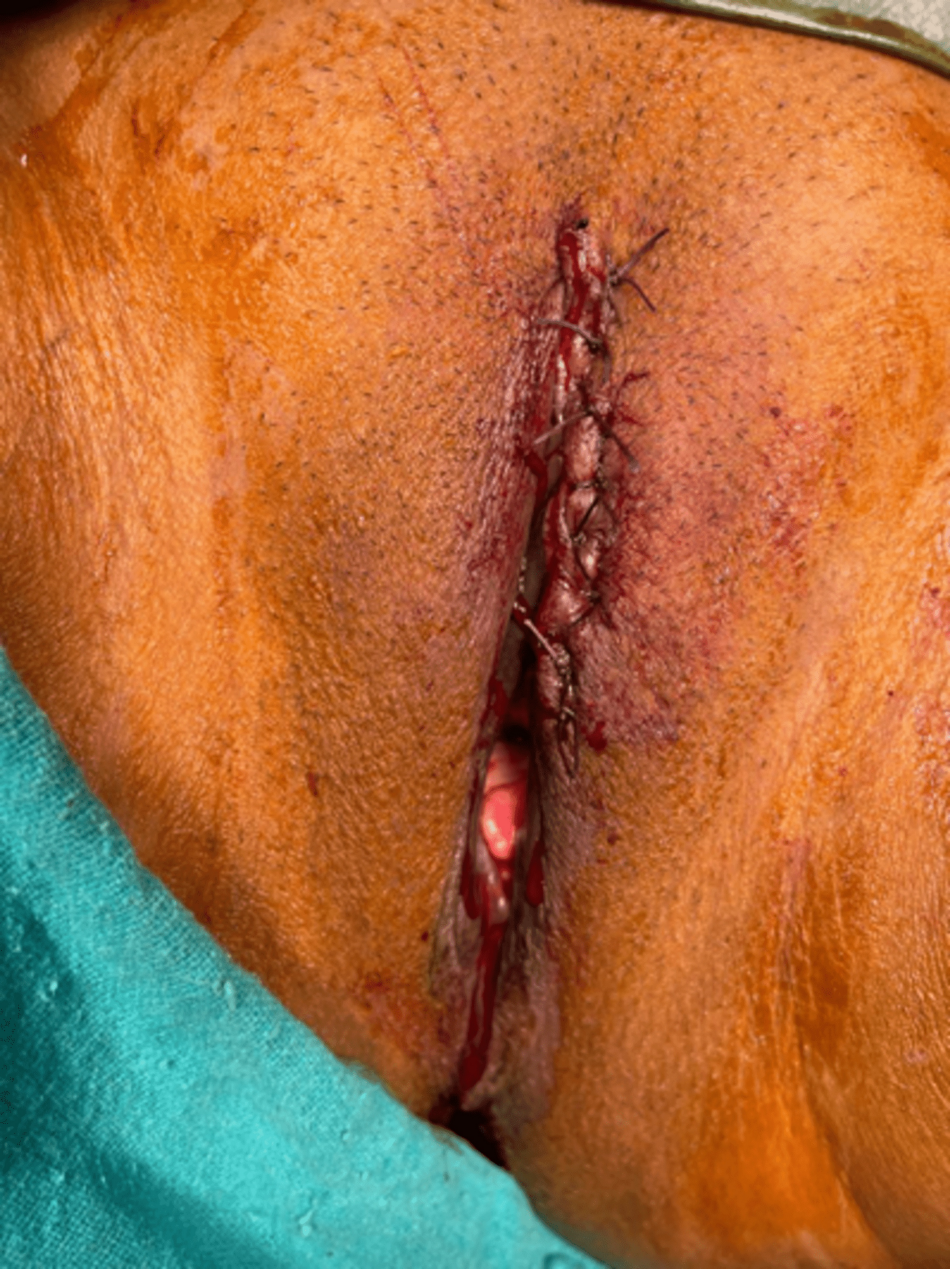
Postoperative status Primary closure was seen after wide local excision.

**Figure 3:**
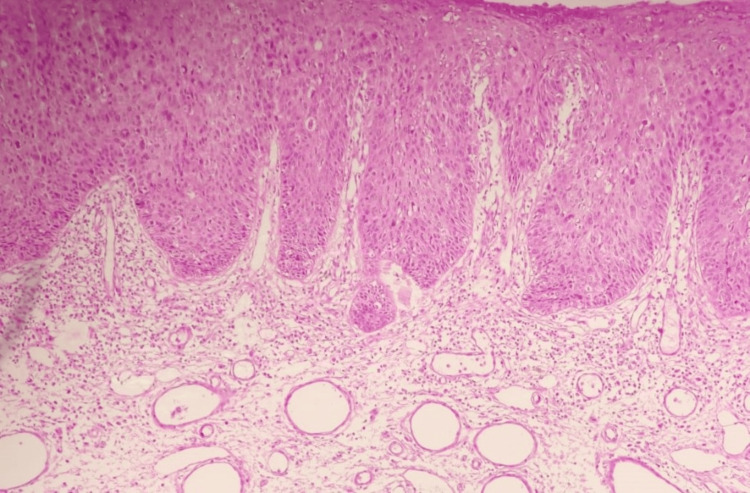
Haematoxylin and eosin stain (H & E) (10X) Full thickness keratinocyte atypia.

**Figure 4:**
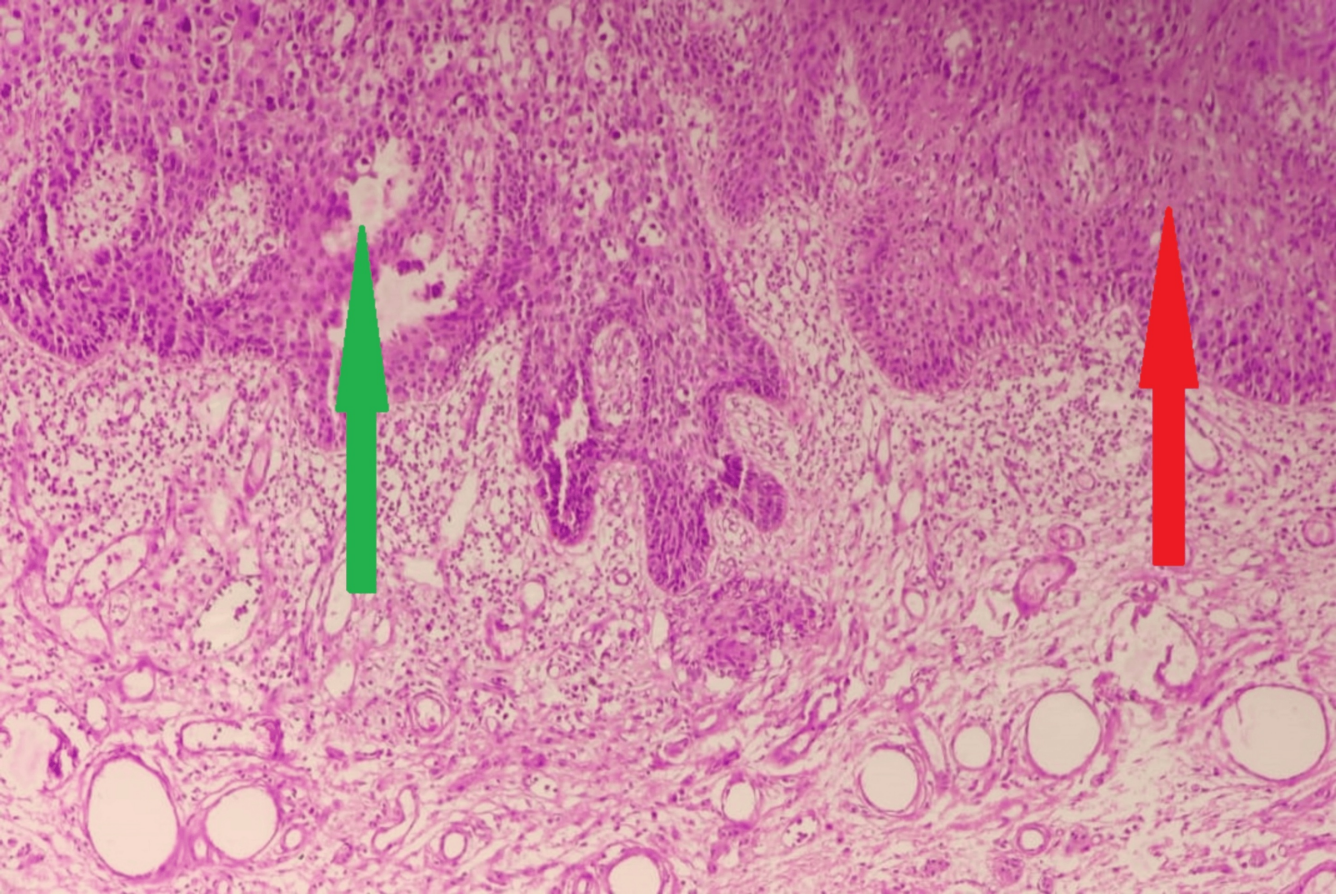
Haematoxylin and eosin stain (H & E) (40X) Full thickness cellular atypia and dyskeratosis (green arrow). Adjacent area shows the normal epidermis (red arrow).

**Figure 5:**
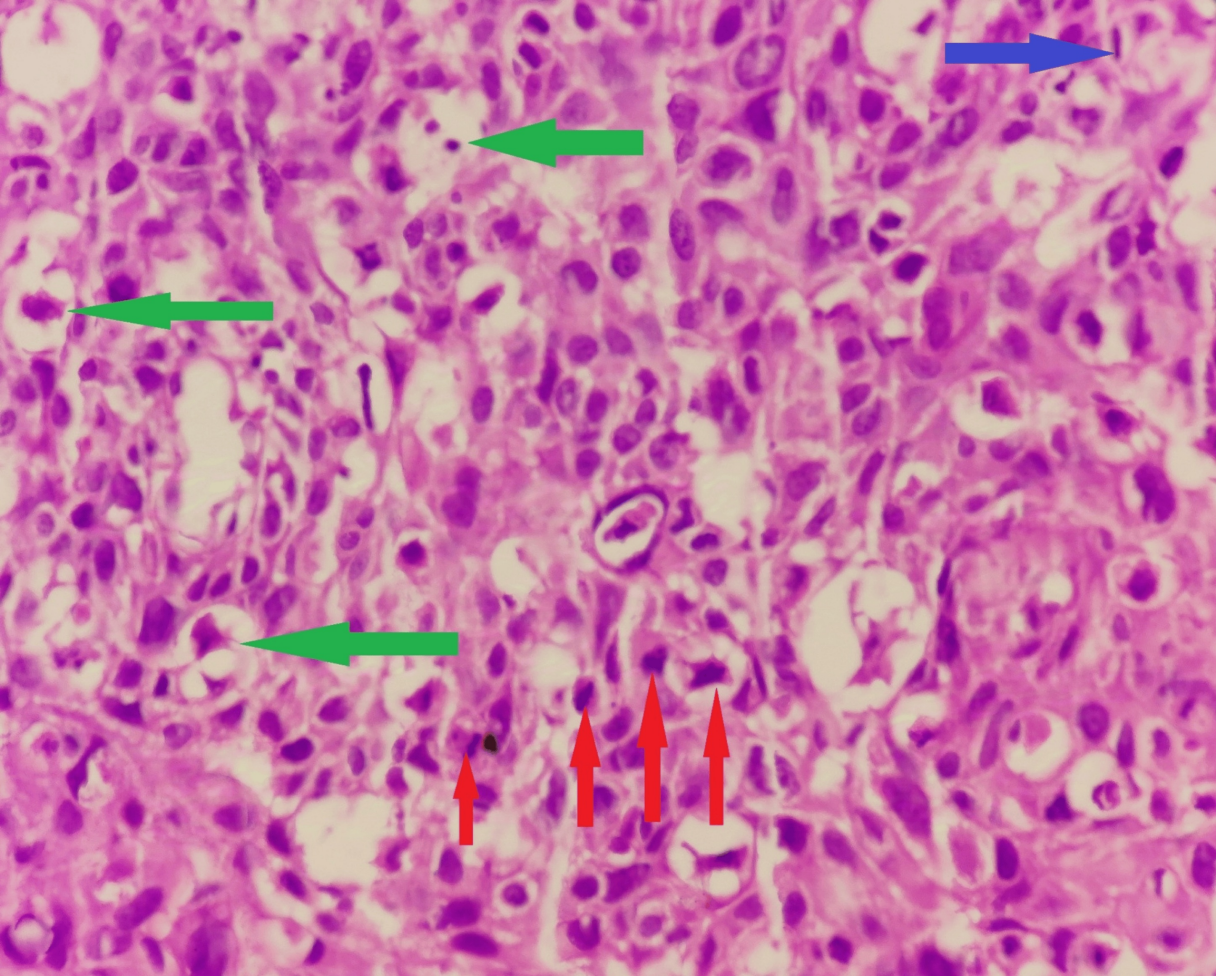
Haematoxylin and eosin stain (H & E) (40X) Dysplastic keratinocytes with marked nuclear atypia and multinucleation (green arrow), mitosis (red arrow), and dyskeratosis (blue arrow).

**Figure 6:**
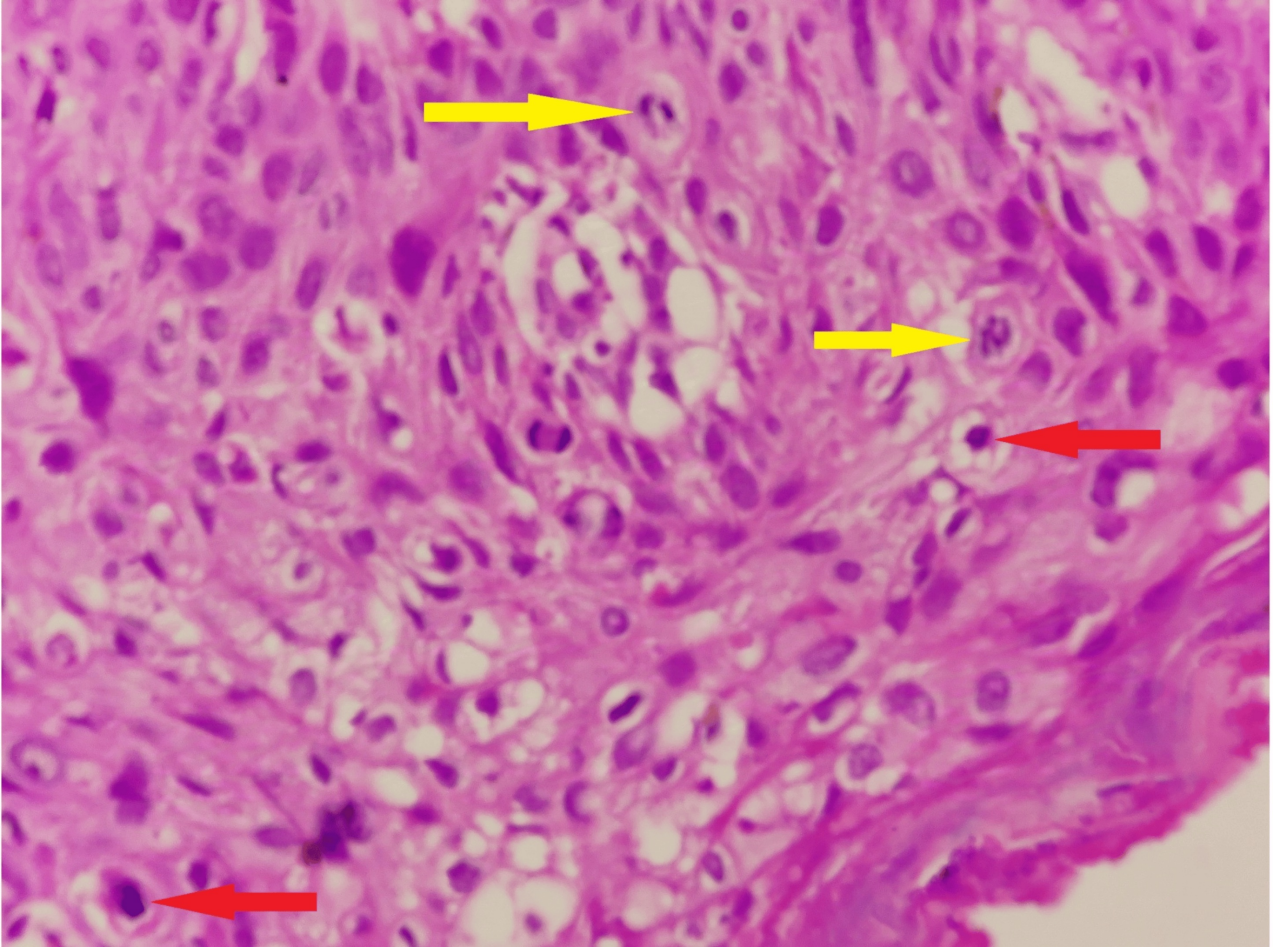
Haematoxylin and eosin stain (H & E) (40X) Dysplastic keratinocytes with marked atypia (red arrow) along with mitosis (yellow arrow).

**Figure 7:**
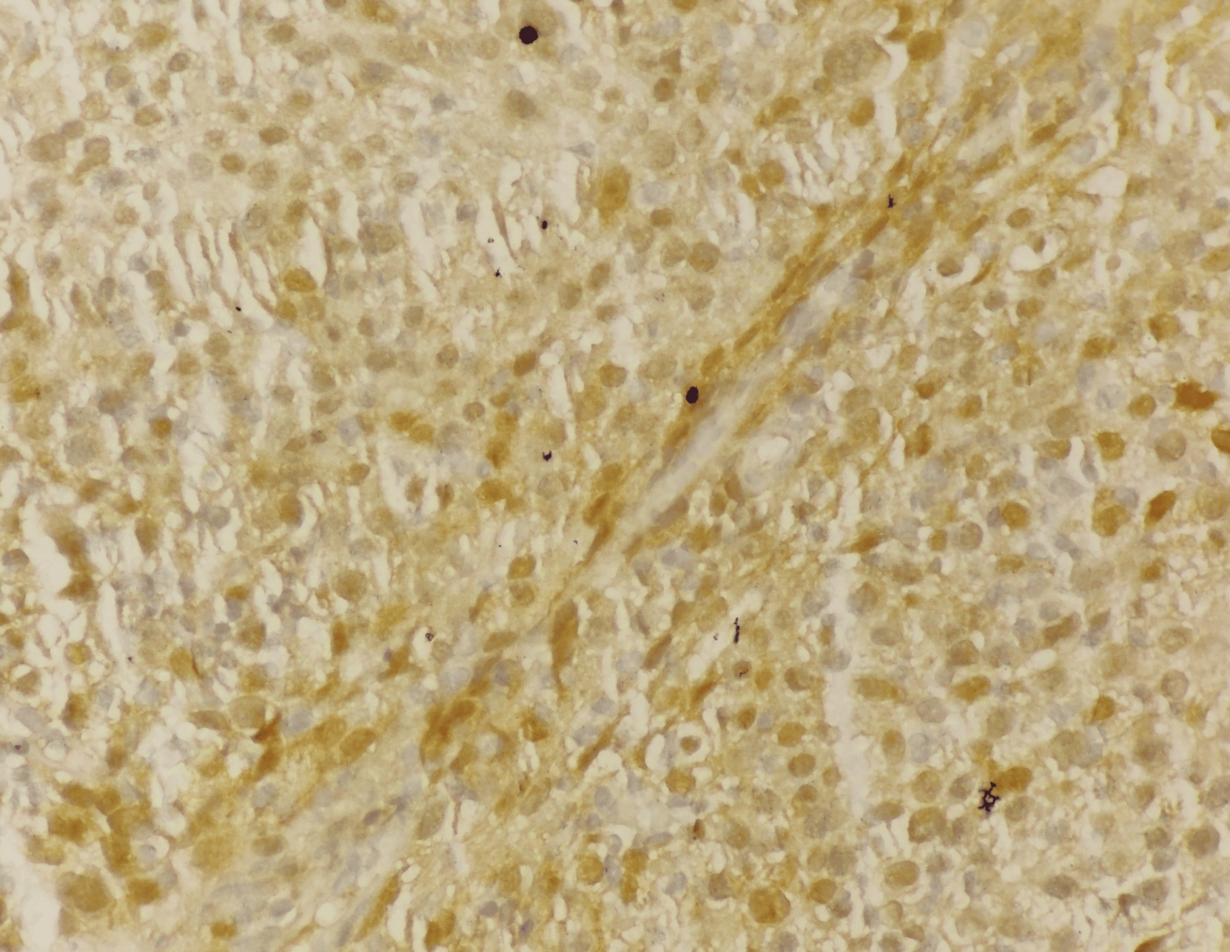
p16 (40X) Strong positive staining of all atypical cells.

A 32-year-old married female presented with a single ulcerative lesion associated with itching and discharge for the last six months on the left upper labial fold. The lesion initially started as a tiny asymptomatic erythematous papule and gradually enlarged into an ulcerative lesion with moderate pruritus and clear ooze. There was no history of associated fever, chills, or any other lesions in the genital area. She had been locally treated with antibiotics and steroids before presenting to our institute but did not show any improvement. Clinical examination revealed a single 4x4 cm round to oval well-defined ulcer (Figure 1), with an erythematous floor, and no fixity to the base. The surrounding skin was unremarkable, and there was no evident inguinal lymphadenopathy. Personal, past, and family history was insignificant. Routine laboratory examinations were within normal limits. An incisional biopsy showed squamous cell carcinoma in situ, and an MRI pelvis showed the lesion to be limited to the subcutaneous tissue. Clinically, the differential diagnoses were sexually transmitted diseases, syphilis, chancroid, tuberculosis, Paget's disease, and carcinomas. After a thorough workup, a wide local excision with a primary repair, under spinal anesthesia in a lithotomy position, was performed (Figure 2), and the specimen was sent for histopathological examination. Grossly, the specimen received in the department of pathology was a single, skin-covered soft tissue piece measuring 3.3x2.5x1 cm. Overlying skin measured 3.3x2.5 cm and showed an ulcerative area measuring 2.2x2 cm. The ulcerated area had irregular borders and was at a distance of more than 0.2 cm from all the resection margins. The specimen was grossed entirely, and full thickness sections were submitted. This protocol was followed, in view of the lesion suspected to be neoplastic, persistently ulcerative, or exudative. The tissue was processed and stained with haematoxylin and eosin stain. Histopathological examination showed full-thickness keratinocyte atypia (Figure 3), along with cellular atypia, individual cell dyskeratosis (Figure 4), and apoptotic cells. Nuclear atypia and mitotic figures (Figures 5-6) were also noted in the sections studied. Altered maturation was seen; however, some surface keratinization and intercellular bridges were still present. The underlying dermis showed variable lymphocytic infiltrate. It also showed strong positive staining for p16 (Figure 7). A diagnosis of Bowen’s disease (squamous cell carcinoma in situ) of the left labia majora was made. A six-month follow-up showed an uneventful healed surgical site.

## Discussion

Bowen’s disease was first described by John Templeton Bowen in 1912, wherein he discussed two rare cases with multiple erythematous plaques over sun-unexposed areas, titled chronic atypical epithelial proliferation [6]. French dermatologist Jean Darier discussed two comparable cases and labeled them as “precancerous dermatosis of Bowen or dyskeratosis lenticularis et discoides.” Over a period of time, this disease was labeled as Bowen’s disease [7,8]. p53 mutation is a major predisposing factor leading to Bowen’s disease, owing to the incremental exposure to UV light, leading to DNA damage and decreased immune status promoting clonal expansion of p53 [9,10]. A strong association with HPV 16 has been documented with vulvar Bowen’s disease [11].

Bowen’s disease typically occurs above the age of 60 years [3]. Some studies have revealed a slight female preponderance [12]. A study in the Caucasian population has revealed a high incidence (1.42/1000) [13]. However, in the Indian population, the exact incidence of Bowen’s disease is eventually not known [14].

A study done by Kossard et al. stated that the most common site for the occurrence of Bowen’s disease is the head and neck region (44%), followed by the lower limb (29.8%), upper limb (19.8%), and torso (6.5%) [15]. In 10%-20% of the cases, multiple sites can be involved [16].

Clinically, a typical Bowen’s disease is a single gradually enlarging erythematous lesion that is well demarcated and may have a scaling or a crusted surface and is usually located in the head and neck region [17]. In our case, the lesion was solitary, itchy, erythematous, and ulcerative in a young female. There was no history of any predisposing or underlying factors. Though few anecdotal cases of Bowen’s disease of genitalia and the perianal area have been described, it still remains a rare site. Clinical examination followed by a biopsy and histopathological examination confirms the diagnosis.

The histopathological differential diagnoses of Bowen’s disease are actinic keratosis, arsenical keratosis, and superficially invasive squamous cell carcinoma. Actinic keratosis occurs in sun-exposed areas, and microscopically, the epidermal dysplasia is not full thickness. Arsenical keratosis is a bullous dermatopathy with a history of long-term exposure to metal arsenic. Microscopically, numerous vacuolated keratinocytes are seen. Superficially invasive squamous cell carcinoma shows invasion. The hallmark of Bowen’s disease is the absence of invasion. Bowen’s disease shows atypical cells with enlarged and pleomorphic nuclei along with an intact basal layer, whereas reactive atypical cells show mild nuclear changes. Pleomorphic changes if present are of mild degree.

A range of therapeutic modalities are available, namely topical chemotherapy, light-based procedures, surgical modalities, and destructive modalities each having its own place in the treatment armamentarium and hence their merits and demerits should be cautiously weighed [3]. As Bowen’s disease usually occurs in geriatric people, frequently in areas with inadequate wound healing, non-invasive treatments are preferred [18]. Surgical treatment is usually preferred in cases of smaller lesions [12,19]. Wide local excision was performed in our case owing to the long-standing lesion resistant to antibiotic and steroid therapy.

Bowen’s disease is a slow-growing premalignant lesion and hence has an excellent prognosis [14]. Spontaneous regression has been reported presumably due to Fas-mediated apoptosis [20]. Recurrence is moderately rare with a reported rate of approximately 6% within five years of taking adequate treatment and usually occurs in immunosuppressed individuals [1]. Progression to invasive carcinoma is one of the sequelae of Bowen’s disease. A six-month follow-up study of our case has not revealed any recurrence.

## Conclusions

The asymptomatic nature, along with the early, nonspecific, varied, and subtle presentations of Bowen’s disease, poses diagnostic challenges even for experienced clinicians. Our case highlights the potential of Bowen’s disease to mimic common dermatological lesions. Hence, a very high index of suspicion aided by histopathological examination is essential for early diagnosis and treatment. There is no single definitive way for all patients, and hence the treatment requires a multimodality approach.
